# Long Non-coding RNA PVT1 as a Prognostic and Therapeutic Target in Pediatric Cancer

**DOI:** 10.3389/fonc.2019.01173

**Published:** 2019-11-06

**Authors:** Ariadna Boloix, Marc Masanas, Carlos Jiménez, Roberta Antonelli, Aroa Soriano, Josep Roma, Josep Sánchez de Toledo, Soledad Gallego, Miguel F. Segura

**Affiliations:** ^1^Group of Translational Research in Child and Adolescent Cancer, Vall d'Hebron Research Institute (VHIR), Universitat Autònoma de Barcelona (UAB), Barcelona, Spain; ^2^Institut de Ciència de Materials de Barcelona (ICMAB-CSIC), Esfera UAB, Cerdanyola del Vallès, Spain; ^3^CIBER de Bioingeniería, Biomateriales y Nanomedicina (CIBER-BBN), Madrid, Spain

**Keywords:** PVT1, lncRNA, epigenetic, 8q32, pediatric cancer

## Abstract

In recent decades, biomedical research has focused on understanding the functionality of the human translated genome, which represents a minor part of all genetic information transcribed from the human genome. However, researchers have become aware of the importance of non-coding RNA species that constitute the vast majority of the transcriptome. In addition to their crucial role in tissue development and homeostasis, mounting evidence shows non-coding RNA to be deregulated and functionally contributing to the development and progression of different types of human disease including cancer both in adults and children. Small non-coding RNAs (i.e., microRNA) are in the vanguard of clinical research which revealed that RNA could be used as disease biomarkers or new therapeutic targets. Furthermore, many more expectations have been raised for long non-coding RNAs, by far the largest fraction of non-coding transcripts, and still fewer findings have been translated into clinical applications. In this review, we center on PVT1, a large and complex long non-coding RNA that usually confers oncogenic properties on different tumor types. We focus on the compilation of early advances in the field of pediatric tumors which often lags behind clinical improvements in adult tumors, and provide a rationale to continue studying PVT1 as a possible functional contributor to pediatric malignancies and as a potential prognostic marker or therapeutic target.

## Pediatric Cancer

Pediatric cancers are rare diseases which differ from adult malignancies owing to their etiology, biology, response to treatment, and outcome. The most frequent adult cancers are of epithelial origin and, in some cases, are caused by environmental factors. By contrast, pediatric tumors tend to be of hematologic, mesenchymal, or nervous system origin and their etiology is often little known (1). Approximately 160,000 cases of cancer in children and adolescents are diagnosed every year worldwide, accounting for up to 2% of all cancers (2). In recent decades, with the introduction of multimodal treatments, the outcome of children and adolescents has improved, reaching ~80% of overall survival. Despite this significant improvement, cancer remains the leading cause of death in children, and adolescents worldwide (3). Furthermore, two thirds of patients may suffer severe side effects associated with these intensive treatments (4). Thus, the development of safe and more effective therapies is a must.

## LncRNAs and Cancer

### The Emerging Opportunity of Long Non-coding RNAs (LncRNAs)

Current therapies are directed at targeting the functionality of the human translated genome (i.e., proteins), barely 2% of all transcribed genetic information (5). Therefore, use of the largest part of the transcribed genome for therapeutic, diagnostic, and prognostic purposes remains unexplored (6). Recent advances in high-throughput sequencing technologies have been crucial for improving understanding of non-coding RNA (ncRNA), which represents a significant fraction of all transcribed RNAs, and in the past, were considered evolutionary junk or transcriptional noise (7, 8). However, ncRNA are now known to be important regulators of biological processes such as gene expression (9). Furthermore, interest in ncRNAs has grown owing to their implication in different diseases, such as cancer (6, 10).

In general, ncRNAs are classified according to their length as small ncRNAs (sncRNAs) or long ncRNAs (lncRNAs), smaller than 200 nt and larger than 200 nt, respectively (11).

### Functions of LncRNAs

LncRNAs functionality depends on their subcellular distribution (12). LncRNAs located in the nucleus can regulate gene expression at different levels: (a) LncRNAs can interact with transcription factors or chromatin-remodeling complexes to regulate gene expression in *cis*, i.e., when the lncRNAs locus is proximal to the regulated gene, or in *trans*, when the gene affected and lncRNAs are at distant locations of the genome (13). For example *ANRIL* is a lncRNA that interacts with the polycomb repressive complex-1 (PRC1) and−2 (PRC2) and mediates transcriptional silencing of *INK4b-ARF-INK4a* locus. Specifically, ANRIL binds to Suz12 to recruit PRC2 complex which initiates H3K27me3, a post-translational histone mark indicative of transcriptional repression (14); (b) LncRNAs may bind directly to DNA, causing chromatin remodeling or looping, where the lncRNAs can recruit chromatin modifiers (e.g., histone methyltransferases, DNA methyltransferases) to enhance or repress gene transcription (15). In fact, there are evidence of sequence-specific interactions of lncRNAs with DNA via triple-helix (triplex) formation, a structure that allows lncRNAs to recruit protein complexes to specific genomic regions and regulate gene expression. For example, the GATA6-AS lncRNA regulates the expression of several genes related to cardiac development using this mechanism (16); (c) finally, other functions of lncRNAs such as their participation in splicing and export or translation of mRNA have also been described (17). One of these examples is the lncRNA Fas-antisense or Saf, which interacts with Fas receptor pre-mRNA and with the human splicing factor 45 (SPF45) in the nucleus of the cell. As a consequence, the exclusion of exon 6 from the Fas mRNA is produced. The resulting protein product is a soluble Fas that protects cells against FasL-induced apoptosis (18).

When lncRNAs are in cytoplasm, they can interact with proteins, thereby either activating or inhibiting its function (19). For example, lincRNA-21 is able to interact with the Heterogeneous Nuclear Ribonucleoprotein K, and modulates the P53 transcriptional response (20). Furthermore, lncRNAs may also regulate mRNA stability and translation processes. For instance, the TMPO-AS1 lncRNA regulates the expression of Thymopoietin, a protein involved in the maintenance of the nuclear envelope structure. Qin et al. demonstrated that the knockdown of TMPO-AS1 also resulted in a decrease of TMPO mRNA and protein levels (21).

However, one of the most described roles of lncRNAs in cytoplasm is microRNA (miRNA) sequestration. LncRNAs may contain multiple miRNA binding sites and can sequester them, thereby reducing their availability for their target genes (22). In particular, lncRNAs may regulate the expression of a certain mRNA by having a miRNA recognition element (MRE) in its sequence. This competition for a miRNA gives name to the competing endogenous RNA (ceRNA) hypothesis formulated by Salmena et al. (23). One of such examples is the lncRNA FER1L4, which modulates PTEN expression by sponging miR-106a-5p in gastric (24) or in colon cancer (25).

LncRNAs present evolutionarily-conserved secondary structure and function, although their exon sequence is much less conserved than protein-coding genes (8, 26). LncRNAs have been reported to present tissue-specific expression and their deregulation has been associated with cancer development, metastasis, and patient outcome (13) often by regulating oncogenes or tumor suppressors.

For example, one of the most studied lncRNAs is the Hox transcript antisense intergenic RNA (HOTAIR). HOTAIR, has been shown to be overexpressed in multiple human tumors such as breast, pancreatic, liver, lung, and hepatocellular cancer among others, and has been involved in different hallmarks of cancer (6, 27). Mechanistically, HOTAIR acts as a scaffold for the chromatin repressors polycomb repressor complex 2 (PRC2) and histone lysine-specific demethylase 1 (LSD1) and may perform a *cis* and *trans*-regulation of HOX genes and HOXD cluster, respectively (28). While the bibliography is abundant for tumors in adult patients, examples are scant in the field of pediatric cancer. One such example is the lncRNA neuroblastoma-associated transcript 1 (NBAT-1) which was associated with good prognosis in high-risk neuroblastoma patients (29). Mechanistically, NBAT-1 epigenetically controls the expression of target genes involved in cell proliferation, differentiation and cell invasion by interacting with EZH2, a subunit of PRC2 (29).

## Plasmacytoma Variant Translocation 1 (PVT1)

One of the lncRNAs that is attracting attention in both pediatric and adult tumors is the plasmacytoma variant translocation 1 (PVT1). Initially described in the middle 80's, this gene was identified as a breakpoint site in chromosome 6;15 translocations, which are associated with murine plasmacytomas (30). In humans, this kind of translocation occurs in rare variants of Burkitt's lymphoma (31). Herein, we focus on this complex and multi-functional lncRNA to raise awareness and pave the way toward better characterization of its use as a potential prognostic marker or therapeutic target in pediatric cancer.

### PVT1 Gene Structure

**Figure 1:**
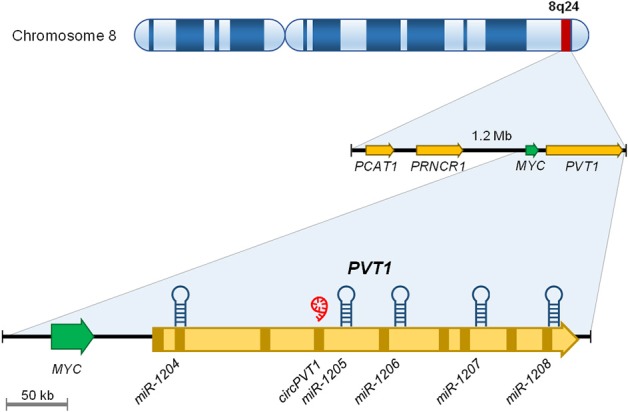
Graphical representation of the 8q24 genomic locus.

“Plasmacytoma variant translocation 1” or PVT1 is a long intergenic ncRNA encoded in the human *PVT1* gene (Figure 1). PVT1 gene is located at the chromosomal locus 8q24.21 which contains the *MYC* oncogene. This region is frequently altered in many types of cancer owing to chromosomal translocations, amplifications or deletions, single nucleotide polymorphisms (SNP) and/or viral integrations (32, 33). The PVT1 gene contains nine exons, which produce multiple transcripts between 2.7 and 3.3 kb in length by alternative splicing (32). Thus, far, none of these transcripts have been shown to be translated into a protein product (32).

In addition to these splicing variants, *PVT1* contains a cluster of five different microRNA (i.e., miR-1204, miR-1205, miR-1206, miR-1207, and miR-1208) which are encoded in the intronic regions of PVT1, except miR-1204, which is located in exon 1b (32, 34). Furthermore, PVT1 encodes a circular RNA, which is presented as a covalent closed loop structure without polyadenylated tail (35, 36). This structure, indeed, also has the capacity to sequester miRNAs such as miR-497 (37, 38) or miR-125 (39, 40).

### PVT1 Participates in Embryonic Development

Pediatric cancers are frequently originated in developing organs where the integration of multiple signaling pathways related to proliferation, growth factor signaling, developmental angiogenesis and programmed cell death, take place (41). It is in this scenario where lncRNA such as *PVT1* become critical for proper physiologic development (42). One of such examples is a recent report showing that *PVT1* is involved in the early stages of development, such as pre-implantation and oocyte maturation (43). Moreover, the loss of PVT1 function may also be implicated in human disease. For example, PVT1 expression was found to be significantly lower in pregnant women with preeclampsia than that in women with normal pregnancies. Loss of function experiments in trophoblast cell lines showed that PVT1 silencing resulted in cell proliferation inhibition and apoptosis induction. Conversely, ectopic expression of PVT1 increased trophoblastic cell proliferation (44).

In following steps of embryonic development, PVT1 has also been shown to play a significant role in neuronal differentiation. Apparently, *PVT1* expression is high in pluripotent stem cells, whereas it decreases during neuronal differentiation (45).

### Functional Role of PVT1 in Cancer

Several studies reported that PVT1 is overexpressed in a wide variety of cancers compared to non-tumoral tissues, including breast and ovarian cancer (46), pancreatic ductal adenocarcinoma (47), cholangiocarcinoma (47), thyroid carcinoma (47), malignant pleural mesothelioma (47), pediatric malignant astrocytoma (46), acute myeloid leukemia (46), and Hodgkin's lymphoma (46). Less evidence, however, also supports a potential role of PVT1 as a tumor suppressor (48).

PVT1 may impact on tumor biology through different mechanisms:

#### Role of the miRNA Cluster

PVT1 encodes a cluster of five miRNA (miR-1204, miR-1205, miR-1206, miR-1207, and miR-1208) (34) which have been shown to play both oncogenic and tumor-suppressive roles. For example, miR-1204 has been shown to be overexpressed and associated with poor prognosis in breast cancer (BC). The ectopic expression of miR-1204 promoted proliferation, epithelial to mesenchymal transition and invasion of BC cells by targeting the vitamin D receptor (49). MiR-1204 was also shown to promote cell proliferation in different types of cancer such as ovarian squamous cell carcinoma (50), non-small cell lung cancer (51), or in hepatocellular carcinoma (52). In line with these results, miR-1205 expression was found to be increased in primary prostate tumors and castration-resistant prostate cancer cell lines. Functional analyses revealed that miR-1205 induced cell proliferation and cell cycle progression (53). Concurring with these results, miR-1205 inhibition impaired osteosarcoma proliferation *in vitro* and *in vivo* by suppressing Wnt/β-catenin activation (54). Overexpression of miR-1207-5p has been shown to promote cancer stem cells traits in ovarian cancer cells by targeting inhibitors of the Wnt/β-catenin pathways (55) and induce breast cancer cell proliferation by targeting STAT6 (56).

Conversely, tumor suppressive roles for some of these miRNAs have also been reported. For example, ectopic expression of miR-1205 significantly inhibited tumor growth in NSCLC mouse xenograft by targeting MDM4 and E2F1. In addition, multiple circRNA have been suggested to sponge miR-1205 in glioma (57–59) and papillary thyroid cancer (60) to increase oncogenicity of these tumors. MiR-1207-5p inhibition induced stem phenotype and expression of stem cell markers, such as OCT4, C-MYC, and SOX2, in nasopharyngeal cancer (61). MiR-1207-5p also has shown to reduce cell invasion *in vitro* and metastasis in a lung cancer mouse xenograft model (62).

#### PVT1-MYC Interaction

The proximal location between the well-established oncogene MYC and PVT1 in the 8q24 region prompted researchers to analyze whether this lncRNA may somehow regulate or be regulated by MYC, a master regulator of cell growth, proliferation, and differentiation, widely implicated in cancer (47). In fact, genomic co-amplification of MYC and PVT1 has been reported in different types of tumor (63). The first evidence found for the interaction between these two molecules was reported by Carramusa et al. who found that PVT1 can be transcriptionally regulated by MYC proteins (64). Further in depth studies revealed that PVT1 interacts with MYC in the nucleus and inhibits its degradation by blocking MYC phosphorylation at threonine 58 (65). Moreover, stabilized MYC protein can activate a positive feedback loop, thereby activating PVT1 expression. Hence, MYC and PVT1 enhance each other's oncogenic effects (65). Concurring with these observations, siRNA-mediated silencing of PVT1 resulted in a reduction in MYC protein levels in different tumor types such as colorectal carcinoma, breast, and ovarian cancer (33). Another level of regulation is also possible, since *MYC* can be targeted by miR-1205, one of the miRNA encoded in the PVT1 RNA (66). Other authors, however, suggested that the oncogenic activities of MYC and PTV1 are independent of each other (67).

#### PVT1 as a Mediator of the Tumor Suppressor Role of p53

Since p53 modulates the expression of several coding and non-coding genes, Barsotti et al. analyzed a potential relationship between p53 and *PVT1*. Experimental evidence demonstrated that *PVT1* contains several p53-binding elements and that certain stress-inducing agents such as DNA-damaging drugs, induced p53-dependent expression of *PVT1*. The authors went further and found mature levels of miR-1204 to be raised after p53 activation and that it was one of the mediators of the tumor-suppressive functions of p53 in colon cancer cell lines (46).

#### PVT1 as a Competing Endogenous RNA (ceRNA)

ceRNA are transcripts that may contain multiple miRNA binding sites (alternatively known as mRNA response elements) that regulate gene expression by sequestering miRNA which otherwise would be bound to their target mRNA. Several studies reported that ceRNA are key regulators of cancer progression (68, 69). Several miRNA have been reported to interact directly with PVT1 in different cancers (Table 1) and, importantly, restoration of miRNA levels partially rescues the oncogenic effect of PVT1 overexpression (40, 102).

**Table 1:** Examples of PVT1-interacting microRNAs.

**Type of cancer**	**Effects of PVT1 silencing**	**MiRNA**	**Targets**	**References**
Bladder cancer	↓ Tumor growth	miR-31-5p	CDK1	(70)
	↓ Tumor growth	miR-128-3p	VEGFC	(71)
Cervical cancer	↑ Cell apoptosis	miR-195-5p	SMAD3	(72)
Colorectal cancer	↓ Tumor growth	miR-455-5p	RUNX2	(73)
	↓ Cell proliferation, invasion, and migration	miR-26b	-	(74)
ESCC	↓ Tumor growth	miR-203a-3p	LASP1	(75)
Hepatocellular carcinoma	↓ Tumor growth	miR-150-5p	HIG2	(76)
	-	miR-365-3p	ATG3	(77)
	↓ Cell proliferation and invasion	miR-186-5p	YAP1	(78)
HUVEC^*^	↓ Cell migration	miR-26b-5p	ANGPT2 and CTGF	(79)
Gallbladder cancer	↓ Cell proliferation and invasion	miR-143-3p	HK2	(80)
Gastric cancer	-	miR-152-3p	CD151, FGF2	(81)
	-	miR-216a-5p	YBX1	(82)
	↓ Cell proliferation and migration	miR-186-5p	-	(83)
Glioma	↓ Tumor growth	miR-190a-5p	MEF2C	(84)
	↓ Tumor growth	miR-488-3p		
	↓ Invasion and migration	miR-200a-3p	-	(85)
	↓ Tumor growth	miR-128-3p	GREM1	(86)
	↓ Cell proliferation	miR-186-5p	-	(87)
Lung cancer	↓ Cell proliferation	miR-126-3p	SLC7A5	(88)
LSCC	↓ Cell proliferation and migration	miR-519d-3p	-	(89)
Melanoma	↓ Cell proliferation	miR-26b-5p	-	(90)
NSCLC	↓ Tumor growth	miR-195-5p	-	(91)
	↓ Tumor growth	miR-497-5p	-	(92)
	↓ Cell proliferation	miR-200a-5p	MMP9	(93)
		miR-200b-5p		
	-	miR-216b	Beclin 1	(94)
	↓ Cell proliferation and invasion	miR-125b-5p	E2F2	(40)
Osteosarcoma	↓ Tumor growth	miR-195-5p	-	(95)
	↑ Chemoresistance to gemcitabine	miR-152-3p	c-MET	(96)
	↓ Cell proliferation	miR-497-5p	HK2	(97)
Ovarian cancer	↓ Cell proliferation, invasion, and migration	miR-133a-3p	-	(98)
	↓ Cell proliferation	miR-140-5p	-	(99)
Pancreatic cancer	-	miR-488-3p	-	(100)
	↓ Cell proliferation, invasion and migration	miR-448	SERBP1	(100)
	↓ Tumor growth	miR-20a-5p	ULK1	(101)
PTC	↓ Cell proliferation and invasion	miR-30a-5p	IGF1R	(102)
Prostate cancer	↓ Cell proliferation and migration	miR-186-5p	Twist1	(103)
Renal cell carcinoma	↓ Cell proliferation and invasion	miR-16-5p	-	(104)
Retinoblastoma	↓ Tumor growth	miR-488-3p	Notch2	(105)

*ESCC, Esophageal squamous cell carcinoma; LSCC, Laryngeal squamous cell carcinoma; NSCLC, Non-small cell lung cancer; PTC, Papillary thyroid carcinoma; HUVEC, Human umbilical vein endothelial cells*.

* *Indicates that experiments were performed in non-tumoral human cell lines*.

## *PVT1* and Pediatric Cancer

Very little is known on the molecular mechanisms that could alter PVT1 expression in pediatric tumors. While genetic aberrations at the 8q24 locus could be responsible for deregulated PVT1 levels in adult malignancies, few cases have been described in pediatric tumors. For example, recurrent translocations of *PVT1-MYC* or *PVT1-NDRG1* were identified in a large study on medulloblastoma, the most malignant brain tumor in children (106), or genomic amplifications of MYC/PVT1 in pediatric gliomas (107). Additional genetic alterations have been reported in the St. Jude PeCAn Data Portal. The analysis of the *PVT1* gene in a cohort of 3,769 pediatric cancer samples from the projects PCGP (St. Jude-WashU Pediatric Cancer Genome Project) (108) and TARGET (Therapeutically Applicable Research to Generate Effective Treatments) (109) reveals 62 different intronic single nucleotide variants and small insertions and deletions, 8 copy neutral loss of heterozygosity, 50 copy number variants, 43 DNA structural variants, and 6 RNA-seq fusions. However, the clinical relevance of these alterations remains to be determined.

**Figure 2:**
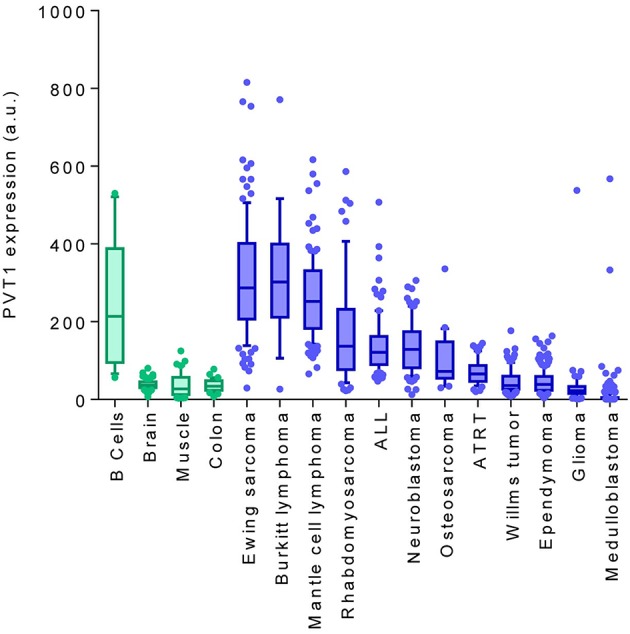
PVT1 expression comparing healthy tissues (green) with pediatric tumors (blue). *PVT1* RNA expression levels were obtained from publicly-available Affymetrix expression array (u133p2) datasets using the “R2: Genomics Analysis and Visualization Platform” software. B cells (GSE12366), brain (GSE11882), muscle (GSE9103), colon (GSE8671), Ewing sarcoma (GSE34620), burkitt lymphoma (GSE26673), mantle cell lymphoma (GSE93291), rhabdomyosarcoma (GSE66533), ALL (GSE68720), neuroblastoma (GSE16476), osteosarcoma (GSE14827), ATRT (GSE70678), wilm's tumor (R2 ID: ps_avgpres_wilmsocg125_u133p2), ependymoma (GSE64415), glioma (GSE19578), medulloblastoma (R2 ID: ps_avgpres_mb500affym223_u133p2). a.u, Arbitraty units.

Regardless of these examples, transcriptomic analysis of *PVT1* showed it to be expressed in different pediatric tumors, with higher levels corresponding to hematologic malignancies and sarcoma, and more moderate expression in nervous system tumors (Figure 2), thereby suggesting that PVT1 has the potential to be used as a prognostic marker or as a therapeutic target in some pediatric tumors. Further studies, however, are needed to reveal the mechanism(s) controlling *PVT1* expression in childhood tumors.

### Prognostic Value of *PVT1* in Pediatric Cancer

While mounting evidence suggests that PVT1 could be a prognostic marker in several adult malignancies (110), very few analyses have been carried out in pediatric tumors. Indeed, Song et al. analyzed *PVT1* expression in osteosarcoma (OS), the most common malignant bone tumor in children, adolescents and young adults. The authors found *PVT1* to be overexpressed in OS cell lines and in tumor samples compared with healthy tissue. Furthermore, high *PVT1* RNA levels correlated with poor overall survival (97).

**Figure 3:**
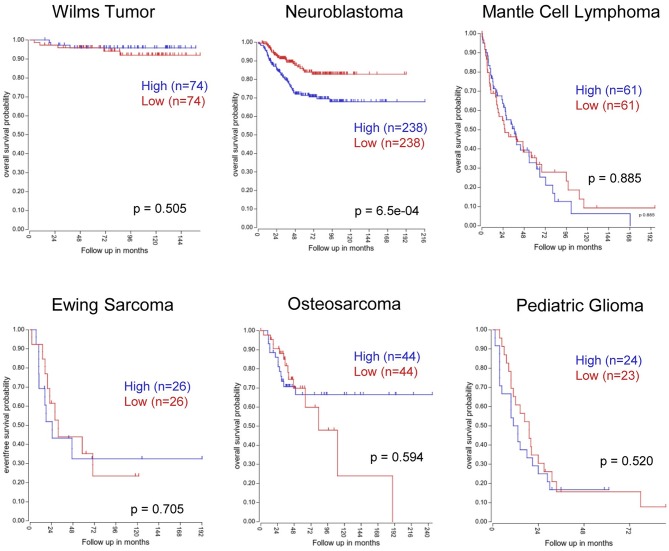
Kaplan-Meier survival curves based on *PVT1* expression in different pediatric tumors. Kaplan-Meier plots were generated using the “R2: Genomics Analysis and Visualization Platform” software. Patient samples were split according to high (above median) or low (below median) PVT1 expression levels from the following datasets: Wilm's tumor (*n* = 148, R2 ID:ps_avgpres_wilmsocga148_u133a), neuroblastoma (*n* = 476, GSE45547), mantle cell lymphoma (*n* = 122, GSE93291), Ewing's sarcoma (*n* = 52, GSE17679), osteosarcoma (*n* = 88, GSE42352), and pediatric glioma (*n* = 47, SE19578).

In line with these observations, we sought to determine whether PVT1 could also be a prognostic marker in other pediatric cancers. Data mining of publicly-available databases which contain PVT1 expression along with annotated clinical parameters was performed for wilm's tumor (*n* = 148, R2 ID:ps_avgpres_wilmsocga148_u133a), neuroblastoma (*n* = 476, GSE45547), mantle cell lymphoma (*n* = 122, GSE93291), ewing's sarcoma (*n* = 52, GSE17679), osteosarcoma (*n* = 88, GSE42352), and pediatric glioma (*n* = 47, SE19578), and revealed that, in neuroblastoma, the expression of PVT1 could have prognostic value (Figure 3). In addition to PVT1 full RNA, other authors used circular RNA (circRNA) of PTV1 as a potential biomarker. CircRNA are uncapped RNA molecules characterized by a covalently closed loop structure without a 3′ polyadenylated tail (111). Kun-Peng et al. found circPVT1 to be upregulated in OS tumors compared with normal tissues. Moreover, higher circPVT1 levels were found in chemoresistant tumors and, furthermore, high levels of circPVT1 were associated with poor overall survival. Finally, those authors demonstrated that the expression of circPVT1 in serum had better sensitivity and specificity than currently-used OS markers such as alkaline phosphatase (112). Concurring with these observations, high levels of circPVT1 (but not total PVT1) were found to be overexpressed in acute lymphoblastic leukemia (ALL) compared to normal bone marrow samples (113); however, their potential to predict patient outcome remains to be determined.

### PVT1 as a ceRNA in Pediatric Tumors

The most common oncogenic attribute of lncRNA is their capacity to sequester tumor-suppressive miRNA. Some examples of *PVT1* have already been described in adult and pediatric malignancies (Table 1). For example, Song et al. showed that PVT1 acts as a sponge to repress miR-497-5p in osteosarcoma cells. In fact, silencing PVT1 resulted in upregulation of miR-497-5p levels which, in turn, target hexokinase 2 (HK2), a key metabolic enzyme that regulates glucose metabolism. Conversely, overexpression of PVT1 reduced miR-497-5p levels and HK2 was upregulated, thereby contributing to enhanced glycolysis, proliferation and motility (97). Similar observations were reported for the interaction of miR-195-5p and *PVT1* also in osteosarcoma. In that case, the authors demonstrated that *PVT1* silencing resulted in increased migration, invasion potential and cell survival, effects that were partially mediated by miR-195-5p (95). Interestingly, miR-195-5p and miR-497-5p are in the same cluster and belong to the same family, i.e., are potentially regulators of the same biological processes.

Finally, a recent study identified PVT1 as an important contributor to resistance to gemcitabine, a nucleoside analog currently used for the treatment of osteosarcoma. Sun et al. showed that PVT1 interacts and inhibits the function of miR-152-3p, thereby promoting increased resistance to gemcitabine by enhancing the activation of C-MET/PI3K/AKT pathway (96).

These first examples are just the tip of the iceberg, since *PVT1* can potentially bind to many more miRNA and should therefore be considered in future studies. Indeed, mining the data available at the experimentally-validated miRNA-LnRNA interactions of the LncBASE (114), revealed that more than 30 different miRNAs are able to interact with PVT1.

### Potential Use of *PVT1* as a Therapeutic Target in Pediatric Cancer

RNA-based therapies are an emerging alternative to conventional treatments owing, in part, to their potential to target all the transcriptome, thereby expanding the number of potential targets. Indeed, proof of concept experiments which silence *PVT1*, such as those mentioned previously [i.e., osteosarcoma (97) and ALL (113)] have already shown that targeting *PVT1* yielded an anti-tumoral response. RNA interference (RNAi), antisense oligonucleotides (ASO) and genome editing (i.e., CRISPR/Cas9 system) are the only currently-available tools to silence lncRNAs [reviewed in (115, 116)]. Furthermore, the function of PVT1 that needs to be targeted will depend on its subcellular distribution. For example, cytosolic PVT1 could be sensitive to siRNA or shRNA-based strategies, whereas nuclear PVT1 would be more sensitive to ASO. One of the examples in which targeting PVT1 could be a new therapeutic strategy was reported recently. Wu et al. showed that siRNA-mediated silencing of PVT1 caused a marked reduction in cell viability in retinoblastoma cell lines. Furthermore, shRNA-mediated loss of PVT1 function also resulted in a reduced tumor growth *in vivo* (105).

In addition to, or instead of, targeting the whole PVT1 molecule, targeting single components of PVT1 may be necessary. For example, in a tumor context where the miRNA encoded within PVT1 participate in disease progression, the use of anti-miR would be recommended. Alternatively, if the oncogenic function of PVT1 is related to its capacity to bind and sequester miRNA, miRNA restoration therapies using miRNA mimetics would be the best approach (117).

## Conclusions and Future Perspectives

Several indicators suggest that *PVT1* could be a future prognostic and therapeutic target for some pediatric tumors. First of all, *PVT1* is a necessary element for the correct embryonic development and differentiation of certain cellular lineages. Aberrant *PVT1* expression may account for the “undifferentiated” state observed in almost all pediatric malignancies. Second, the mutational burden of pediatric tumors is ~14 times lower than adult tumors (118), which suggests that epigenetic alterations are more likely to participate in tumorigenesis or tumor progression than in adult tumors. Third, the fact that *PVT1* has the capacity to bind and modulate the function of multiple tumor-suppressive miRNA, places *PVT1* as a master regulator of several biological processes regulated by these miRNA. Further safety studies analyzing the impact of a systemic *PVT1* loss of function and finding an appropriate clinical formulation to administer small RNA molecules targeting PVT1, are the next steps in implementing the use of PVT1-based therapies for the treatment of tumors of childhood and adolescence.

## Author Contributions

AB, MM, and RA revised bibliography. CJ designed and created the figures. AS, JR, JS, and SG provided intellectual support and expertise in the field of pediatric cancer. AB, MM, and MS wrote the original manuscript. All authors contributed to the edition and the critical review of the manuscript.

### Conflict of Interest

The authors declare that the research was conducted in the absence of any commercial or financial relationships that could be construed as a potential conflict of interest.
